# Plasticity in Preganglionic and Postganglionic Neurons of the Sympathetic Nervous System during Embryonic Development

**DOI:** 10.1523/ENEURO.0297-23.2023

**Published:** 2023-11-03

**Authors:** April Ratliff, Dobromila Pekala, Peter Wenner

**Affiliations:** Department of Cell Biology, Emory University School of Medicine, Atlanta, GA 30322

**Keywords:** autonomic nervous system, chick embryo, homeostatic plasticity, sympathetic nervous system, synaptic scaling

## Abstract

Sympathetic preganglionic neurons (SPNs) are the final output neurons from the central arm of the autonomic nervous system. Therefore, SPNs represent a crucial component of the sympathetic nervous system for integrating several inputs before driving the postganglionic neurons (PGNs) in the periphery to control end organ function. The mechanisms which establish and regulate baseline sympathetic tone and overall excitability of SPNs and PGNs are poorly understood. The SPNs are also known as the autonomic motoneurons (MNs) as they arise from the same progenitor line as somatic MNs that innervate skeletal muscles. Previously our group has identified a rich repertoire of homeostatic plasticity (HP) mechanisms in somatic MNs of the embryonic chick following *in vivo* synaptic blockade. Here, using the same model system, we examined whether SPNs exhibit similar homeostatic capabilities to that of somatic MNs. Indeed, we found that after 2-d reduction of excitatory synaptic input, SPNs showed a significant increase in intracellular chloride levels, the mechanism underlying GABAergic synaptic scaling in this system. This form of HP could therefore play a role in the early establishment of a setpoint of excitability in this part of the sympathetic nervous system. Next, we asked whether homeostatic mechanisms are expressed in the synaptic targets of SPNs, the PGNs. In this case we blocked synaptic input to PGNs *in vivo* (48-h treatment), or acutely *ex vivo*, however neither treatment induced homeostatic adjustments in PGN excitability. We discuss differences in the homeostatic capacity between the central and peripheral component of the sympathetic nervous system.

## Significance Statement

The autonomic nervous system plays a critical role in the survival and health of an organism and therefore must be tightly regulated, as diseases involving autonomic dysregulation, such as hypertension, have dramatic health consequences. Evidence suggests that challenges to autonomic signaling during prenatal and neonatal periods can affect long-term health. This early developmental critical period when the circuit is first establishing excitability levels is likely to depend on different plasticity mechanisms. It is unknown whether homeostatic plasticity (HP), thought to be critical for the maturation of cellular excitability, is expressed in the developing autonomic nervous system. Here, we are testing the hypothesis that mechanisms of homeostatic plasticity are expressed in preganglionic and postganglionic neurons (PGNs) of the embryonic sympathetic nervous system.

## Introduction

The CNS has an extraordinary capability to adapt to changing environments. Particularly responsive is the autonomic nervous system, which is comprised of parasympathetic and sympathetic nervous system branches. Central neurons of the autonomic nervous system project into the periphery and drive neurons in autonomic ganglia. In turn, these postganglionic neurons (PGNs) innervate peripheral tissues to maintain homeostasis within an organism (Jänig, 2006). Adjustments in autonomic output occur routinely, such as transient changes in vasodilation to maintain body temperature. However, it remains unclear how the autonomic nervous system first establishes the setpoint of the baseline sympathetic tone, thus ensuring peripheral targets are not chronically hyperactive or hypoactive. Prenatal and early neonatal development appear to be particularly sensitive stages for establishing proper sympathetic tone, such that early autonomic disruptions as can occur in preterm birth (Patural et al., 2008; Aye et al., 2018; Schlatterer et al., 2022) are associated with long-term autonomic dysfunction and stress-related neuropsychiatric consequences (Hack et al., 2005; Crump et al., 2011; Mulkey and Plessis, 2018; Haraldsdottir et al., 2019; Mulkey and du Plessis, 2019). These results indicate that early development is a critical period for the maturation of healthy autonomic setpoints (Cerritelli et al., 2021). Critical periods represent distinct developmental windows of plasticity during which circuit excitability can be defined. Little is known about the mechanisms of plasticity in the developing autonomic nervous system, but identifying such mechanisms will advance our understanding of the maturation of autonomic tone.

Homeostatic plasticity (HP) represents a set of mechanisms that regulate excitability and could contribute to establishing the autonomic setpoint. When engaged, HP mechanisms ensure that some feature of neural activity (e.g., firing rate, synaptic strength) is homeostatically recovered following a perturbation. Such mechanisms include compensatory adjustments in synaptic currents and excitability of the cell membrane (Tien and Kerschensteiner, 2018). Synaptic compensations can occur by alterations in neurotransmitter receptors (Turrigiano et al., 1998; Turrigiano, 2012), the driving force for synaptic currents (Gonzalez-Islas et al., 2010), or presynaptic neurotransmitter release (Davis, 2006; Goel and Dickman, 2021; Engisch et al., 2022). Compensatory changes in intrinsic membrane excitability, achieved by adjustments in overall function of ion channels, have also been described (Turrigiano et al., 1994; Desai et al., 1999; Desai, 2003; Wilhelm et al., 2009; O’Leary et al., 2013).

Because HP mechanisms alter circuit excitability and are often expressed during a distinct period of development (Desai et al., 2002; Echegoyen et al., 2007; Hennig et al., 2011; Huupponen et al., 2016; Wen and Turrigiano, 2021; Fitzpatrick and Kerschensteiner, 2023), they could be important for determining the trajectory of autonomic tone during a critical period. It is currently unknown whether cells of the developing autonomic nervous system express these HP mechanisms. Therefore, we tested the hypothesis that HP is expressed in developing sympathetic neurons of the chick embryo, which is amenable to studying HP mechanisms during early development (Wenner, 2020).

Previously, our group identified a rich repertoire of HP mechanisms in motoneurons (MNs) in the embryonic chick following *in vivo* synaptic/activity blockade (Gonzalez-Islas and Wenner, 2006; Wilhelm et al., 2009; Wenner, 2020). Here, we hypothesized that sympathetic preganglionic neurons (SPNs) would also express HP mechanisms. We found evidence suggesting homeostatic synaptic plasticity was triggered in SPNs following a 2-d, *in vivo* reduction of GABAergic neurotransmission, which is excitatory at this developmental stage (Wenner and Pekala, 2022). Embryonic SPNs increased synaptic driving force for GABAergic currents in a compensatory fashion by altering intracellular chloride levels, consistent with previous findings in somatic MNs (Gonzalez-Islas et al., 2010; Lindsly et al., 2014). Therefore, HP could play a role in establishing the setpoint for autonomic tone in SPNs. Conversely, reducing synaptic input to PGNs, either chronically *in vivo*, or acutely *ex vivo*, did not induce a response indicative of HP. The presence of HP in SPNs but not in PGNs is considered in the discussion.

## Materials and Methods

### Chicken embryos

All animal procedures were performed in accordance with Emory University’s animal care committee’s regulations. White Leghorn chicken embryos were purchased from Hy-Line Hatcheries North America. Fertilized eggs were kept in the incubator at ∼38°C at 65% relative humidity. Embryos were staged according to the duration of time in the incubator and developmental stages were confirmed using Hamburger and Hamilton (HH) staging (Hamburger and Hamilton, 1951). For the present study, embryos were used from embryonic day ∼3 to 17 (E3–E17, stage 15–43 HH). Sex was not determined in these experiments.

### Tissue isolation

Chick embryo spinal cord tissue was surgically isolated in cooled (14–16°C) Tyrode’s solution containing the following (in mm): 139 NaCl, 12 D-glucose, 17 NaHCO_3_, 3 KCl, 1 MgCl_2_, and 3 CaCl_2_. The solution was oxygenated (95% O_2_/5% CO_2_) throughout the procedure. After isolation was complete, the solution was warmed to 18°C and the tissue was allowed to recover overnight (12–18 h). The following day, spinal cords were transferred to a recording chamber, which was circulated with Tyrode’s solution, oxygenated as above, and held at 27 ± 1°C for electrical recording or imaging (Pekala and Wenner, 2022).

For PGN recordings, lumbosacral (LS) segments of paravertebral chain (LS1–LS7) were isolated from the spinal cord, detaching them at the ventral roots by breaking the gray and white rami, under the same conditions as the cord preparations. However, recordings from this tissue were obtained after 1–2 h recovery period. This short recovery time was feasible as preliminary tests revealed that recordings did not differ between these and overnight preparations.

### Retrograde labeling of SPNs and MNs

**Figure 1.**
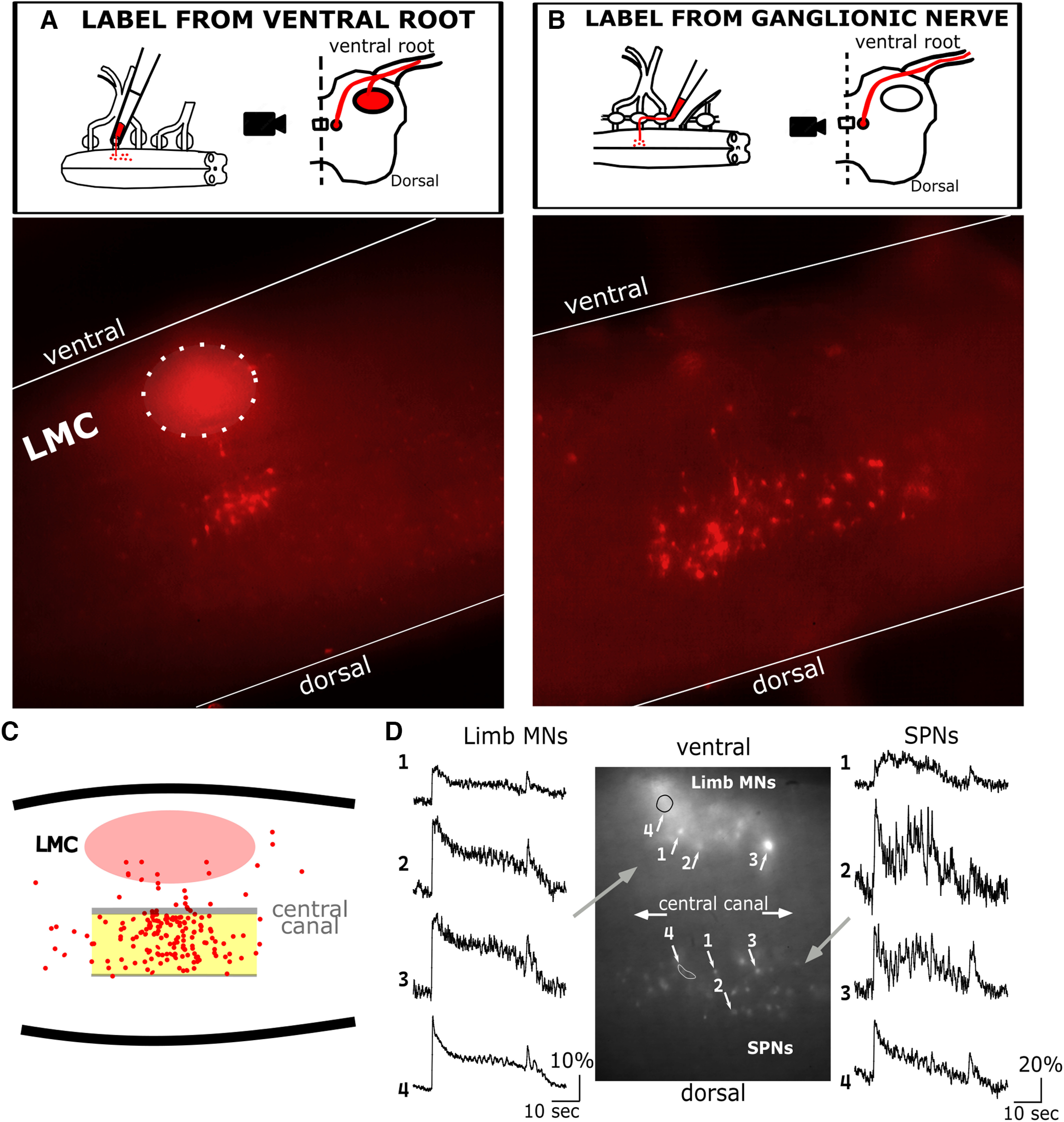
Location and calcium transients of lumbosacral SPNs in E10 embryos. ***A***, Texas Red retrograde labeling from ventral root reveals two populations of neurons. One population is consistent with lateral motor column (LMC), the other population is medial, slightly dorsal of the central canal, consistent with Column of Terni. ***B***, In separate experiments, the medial population of SPNs were also retrogradely labeled from the IGN, which did not result in labeling of limb motoneurons in the LMC. ***C***, Of the 179 total cells imaged on medial surface, 81.0% were in the region between the central canal and halfway to the dorsal edge of the cord (highlighted in yellow), where SPNs reside. ***D***, Calcium transients were observed using cells labeled by the calcium indicator Calcium green. The medial view of the hemicord revealed that both limb MNs and SPNs were active during evoked and spontaneous episodes of network activity (ΔF/F of >20%, *N* = 3 cords).

Embryos were dissected to isolate spinal cords on embryonic day 10 (E10, stage 36 (Hamburger and Hamilton, 1951)). Ventral roots were left intact on the cord, and the cut end of the ventral root was drawn into a suction electrode (at LS1 or LS2) filled with a fluorescent indicator (O’Donovan et al., 1998). For the anatomic examination of SPNs, Texas Red (conjugated with dextran, 10,000 MW, neutral; D1828; Invitrogen) was used. In separate cords, Calcium Green-1 (dextran potassium salt, 10,000 MW anionic, C3713; Invitrogen) was used to visualize calcium transients in both SPNs and MNs. In both cases we used a ratio of indicator to vehicle (H_2_O) of 1 mg:10 μl. In one cord with intact rami, we labeled with Texas Red from the interganglionic nerve (IGN) rather than the ventral root, to confirm the location of the SPN population without contamination of signal from limb MNs (Fig. 1*B*). However, because of the fragility of the white rami, success rates were low with this labeling procedure, so we used the proximal ventral root for labeling in most experiments (*n* = 8).

### Calcium imaging

All images were taken in the medial view of hemisected cords. Regions of interest (ROIs) were drawn around labeled cell bodies to examine changes in fluorescent intensity during episodes of spontaneous network activity (SNA), which are spontaneously occurring synaptic recruitment of the spinal network that can last for over 60 s (Landmesser and O’Donovan, 1984; O’Donovan et al., 2008). After a refractory period of several minutes, another episode of SNA can occur spontaneously, or can be initiated with a brief stimulus pulse of the spinal nerves or spinal cord white matter. Both spontaneous and evoked episodes of SNA were recorded optically by measuring an increase in intensity of fluorescence because of calcium influx in these ROIs (5–15 frames/s). A 60-frame average of a nonlabeled region of the cord was used for subtraction of background fluorescence from each ROI. Each of these background-subtracted ROIs were then normalized by dividing the intensity values in every frame by a 60-frame average of the pre-episode baseline fluorescence for that ROI. This calculation provided ΔF/F, which follows the change in intracellular calcium levels at baseline and during spontaneous or evoked episodes of SNA. These values were measured in both MNs and SPNs. The cell type was determined based on location in the cord using results from retrograde labeling experiments (described above). Cells that were close to the medial surface and located within the appropriate dorsoventral position were classified as SPNs, while cells located in a more lateral position, and on the ventral half of the hemi cord, were considered MNs (Fig. 1).

### Chloride imaging

Embryos were transfected at ∼E3 (stage 15–17 HH) with plasmid containing Clomeleon, a chloride indicator (3.40 μg/μl), in a 1:1 ratio with solution of Fast Green (Sigma-Aldrich) for electrode visualization as described previously (Lindsly et al., 2014). The transfection occurred via injection into the neural tube, followed by lateral electroporation using a train of five square wave pulses of 50-ms duration, at 25 V, at a rate of 1 Hz. At E10, cords were imaged according to previously used procedures (Lindsly et al., 2014) on an Olympus Inverted IX70 microscope. Ratios were calibrated on this system in this previous study which associates a fluorescence resonance energy transfer (FRET)-based ratio of YFP/CFP with a concentration of intracellular chloride. For each cord, both ventral and medial images were acquired using the YFP channel and the CFP channel at 10× magnification. Excitation filter bandwidth was 430–450 nm, the emission filter for the CFP cube was 485 ± 15 nm, or for YFP was 530 ± 15 nm. The dichroic filter cutoff was 460 nm. Neutral density filters were used to reduce light from the arc lamp to 2–6% of full intensity. Images were then processed offline using Simple PCI software. ROIs were drawn around motoneuron cell bodies (ventral view) or presumptive SPN cell bodies within the inclusion criteria (from the medial view of the hemicord), determined by location of retrogradely labeled SPNs (Fig. 1*C*). The intensity of each ROI was background subtracted using a region of the cord without labeled cell bodies, to remove any impact of auto-fluorescence. This process was conducted for both CFP and YFP images, using the same ROIs for both images, and FRET ratio values were expressed as YFP/CFP for each ROI. Cords were excluded from analysis if the coefficient of variation of the FRET ratio values in all ROIs was >0.15, or if the cells had a ratio of <1.2 as this value was associated with cells that were unresponsive in calibration experiments using progressively increasing chloride concentrations in our previous study (Lindsly et al., 2014).

### Extracellular electrophysiology

Extracellular recordings of evoked synaptic activity were conducted using two suction electrodes, one for stimulating the rostral end of the paravertebral ganglia and one for recording at the caudal end. Electrical signals were measured using extracellular amplifiers (AC/DC differential amplifier model 3000, A.M. Systems, filtering parameters for capturing synaptic activity: 300-Hz high pass, 1-kHz low pass). The threshold for inducing a reproducible compound action potential was determined individually for each preparation. This threshold was then tested to determine whether it was sufficient to see synaptic activity. A stimulus intensity of 4× threshold was used for each preparation, as it was sufficient for inducing maximal or near maximal recruitment of preganglionic axons. A stimulus pulse of 0.5-ms duration was delivered through the stimulating electrode at the rostral end of the isolated chain of lumbosacral ganglia. Each sweep was repeated 30–60 times at an interval of 60 s before and after drug perfusion, or after washout. The induced neural activity was quantified by rectifying the voltage trace and normalizing the amplitude of synaptic activity to the amplitude of a prestimulus baseline period. Synaptic discharge was identified by measuring the signal after the SPN orthodromic volley had dropped back to baseline (typically 12–20 ms poststimulus). A period of 10 ms was selected and an average was performed in each sweep to measure the synaptic discharge.

### Intracellular electrophysiology

Whole cell recordings were used exclusively as sharp electrode recordings have been demonstrated to be less reliable for intracellular recordings in PGNs (Springer et al., 2015; McKinnon et al., 2019). These recordings were used to examine the relationship between input current and the output spiking activity, providing a measure of excitability for PGNs. Individual neurons from ganglia between LS1 and LS3 were examined using whole cell patch clamp recordings (Axoclamp 2B, Molecular Devices) to establish various measures of intrinsic excitability. Neurons with resting membrane potential (RMP) more depolarized than −40 were excluded from analysis and cells were held at a holding potential of −70 ± 5 mV (before correction for liquid junction potential). Any sweeps in which cells were not maintained in this range were excluded from analysis. To evaluate excitability, a frequency/current (*f*/I) curve was established by increasing the size of current steps (5-pA intervals, 500-ms duration) in current clamp mode and recording the resulting frequency of action potentials (number/0.5 s). The *f*/I curve procedure was repeated at least three times for each cell and resulting values for each current step were averaged. This procedure was also used to examine additional measures of excitability such as rheobase, threshold voltage, slope of the rising phase of the spike, membrane time constant, peak amplitude, half-width duration, and after-hyperpolarization. Liquid junction potential was measured to be ∼+14 mV. To accommodate for this artificial depolarization, this value was subtracted from voltage measures of RMP and threshold voltage in Table 1.

**Table 1:** Comparison of membrane properties between treatment groups (vehicle and hexamethonium)

Table of membrane properties	Vehicle treated	Hex treated	*t* statistic	*p* value
Capacitance (pF)	25.3 ± 3.5	23.5 ± 4.5	*t*_(15)_ = 0.31	0.76
Resting membrane potential (mV)	−56 ± 2	−59 ± 1.7	*t*_(15)_ = 0.3	0.77
Rheobase (pA)	30.4 ± 4.9	44.7 ± 6.1	*t*_(14)_ = 1.82	0.091
Input resistance (MΩ)	1052.7 ± 115	788.6 ± 110	*t*_(12)_ = 1.72	0.11
Threshold voltage (mV)	−54 ± 1.3	−51 ± 2.4	*t*_(15)_ = −1.19	0.25
Action potential half-width (ms)	4.4 ± 0.5	2.7 ± 0.2	*t*_(13)_ = 2.15	0.051
After-hyperpolarization amplitude (mV)	10.2 ± 1.6	10.5 ± 1.7	*t*_(14)_ = −0.11	0.91
Action potential peak amplitude (mV)	12.6 ± 2.3	24.7 ± 3.5	*t*_(14)_ = −2.94	<0.011*
Membrane time constant τ (ms)	26.8 ± 5.2	18.4 ± 2	*t*_(15)_ = 1.3	0.21
Slope of rising phase (mV/ms)	8.5 ± 3.4	8.1 ± 2.2	*t*_(14)_ = 0.25	0.81

Mean values are listed with standard error. Statistical values reflect the degrees of freedom for each measure compared using a two-tailed, unpaired *t* test. *p* < 0.05 is denoted by *.

### Experimental and statistical analysis

In cases of uneven sampling across preparations, nested sampling techniques were used to estimate effect size and statistical significance, including hierarchical bootstrapping and multilevel modeling. The hierarchical bootstrapping method ensures that each animal is equally represented in the dataset, and data points from the same embryo are not treated as independent from one another. This method was necessary because many other common statistical tests have an intrinsic assumption of independence between all datapoints, as well as homogeneity of variance, and such a dataset would therefore violate these assumptions. Multilevel datasets, such as those which contain repeated measures and multiple cells in a single animal, are thus treated as nested, nonindependent groups when using this bootstrapping method, while the Type-1 error rate is maintained within the appropriately adjusted range to avoid false positives (Aarts et al., 2014; Saravanan et al., 2020). Finally, linear mixed-effect modeling was used (lme4, version 1.1-32, R) to ensure that differences seen between experimental groups were explained primarily by the fixed effect (drug treatment), and that said differences were not explained by random effects such as experimental day or cord number. Statistical test values have been included in figure legends.

### Code availability

Custom programs for data analysis can be provided on request, and can be found publicly available at https://github.com/April-Neuro/SPN-PGN-Analysis.

All animal procedures were performed in accordance with Emory University’s animal care committee’s regulations.

## Results

### Chloride-mediated scaling in preganglionic neurons

To determine whether SPNs have the capacity for HP during embryonic development, we looked at the possibility that these cells express GABAergic synaptic scaling, as this form of plasticity is known to be expressed in somatic MNs of the embryonic chick (Wilhelm and Wenner, 2008; Gonzalez-Islas et al., 2010). GABAergic synaptic scaling is typically demonstrated by recording GABAergic miniature postsynaptic currents (mPSCs), in response to 48-h pharmacological blockade of neurotransmitter receptors or spiking activity and is mediated by trafficking GABA_A_ receptors (GABA_A_Rs) into or out of the synaptic membrane (Kilman et al., 2002; Tien and Kerschensteiner, 2018). We previously demonstrated that GABAergic synaptic scaling in chick embryo MNs was mediated by a different mechanism. GABAergic transmission in embryonic chick spinal neurons is depolarizing and excitatory at these early stages because of elevated intracellular chloride levels (Wenner and Pekala, 2022). We had determined that GABA_A_R blockade led to increases in the intracellular concentration of chloride, thus increasing the driving force for these depolarizing currents (Gonzalez-Islas et al., 2010; Lindsly et al., 2014). One of the ways we showed this was using Clomeleon, a genetically expressed indicator of intracellular chloride (Kuner and Augustine, 2000; Lindsly et al., 2014). In order to test for this mechanism of scaling in SPNs, we first confirmed their location in the spinal cord. Cell bodies of SPNs (*n* = 179) were retrogradely labeled with Texas Red^TM^ from the LS2 ventral root (Fig. 1*A*) or IGN (Fig. 1*B*). Spinal cords were hemisected and imaged from the medial surface of the hemicord. We observed that 81% of labeled cell bodies on the medial surface were located in a region that spanned from the central canal to halfway toward the dorsal edge (Fig. 1*C*). This region is consistent with previous reports of thoracolumbar SPNs (Levi‐Montalcini, 1950; Prasad and Hollyday, 1991) also known as the column of Terni in the chick embryo (Terni, 1923). These criteria were used to isolate the SPNs for Clomeleon labeling experiments.

To determine whether it was possible to evoke scaling in the SPNs, we treated embryos *in ovo* with the GABA_A_R antagonist gabazine (GBZ; Sigma-Aldrich, 10 μm) from E8 to E10 to reduce GABAergic synaptic transmission. This treatment was previously shown to be sufficient to trigger scaling in somatic MNs (Wilhelm and Wenner, 2008), which are similar to SPNs as they are generated from the same precursor population (Stifani, 2014). Spontaneous network activity (SNA) represents a synchronous recruitment of MNs and spinal interneurons which can be measured by a voltage trace or by calcium transient (Landmesser and O’Donovan, 1984; O’Donovan et al., 2008). These episodes of SNA are significantly driven by GABAergic transmission, which is depolarizing at this developmental stage (O’Donovan et al., 1998; Leinekugel et al., 1999; Ben-Ari, 2002). It was unclear, however, whether SPNs were synaptically coupled to the network of neurons which drive episodes of SNA at this early developmental stage. Therefore, we set out to confirm that SPNs also express SNA by measuring the calcium signal associated with this activity. We compared the calcium activity in both MNs and SPNs, which were retrogradely labeled from the ventral root using the calcium indicator Calcium Green (see methods). We found that SPNs exhibited calcium transients during episodes of SNA, in synchrony with somatic MNs, which have been shown to express SNA (Chub and O’Donovan, 1998; O’Donovan et al., 1998; Tabak et al., 2022). Calcium transients (ΔF/F) in labeled cells increased by >20% (*N* = 3 cords) in both electrically evoked and spontaneously occurring episodes of network activity (Fig. 1*D*). This finding confirms that SPNs receive spontaneous synaptic input at this midgestational stage of development, and that SPNs express SNA that is likely driven, in part, by GABAergic synaptic transmission. Thus, blocking GABAergic transmission throughout the cord could trigger GABAergic synaptic scaling in the SPNs, as occurs in somatic MNs.

**Figure 2.**
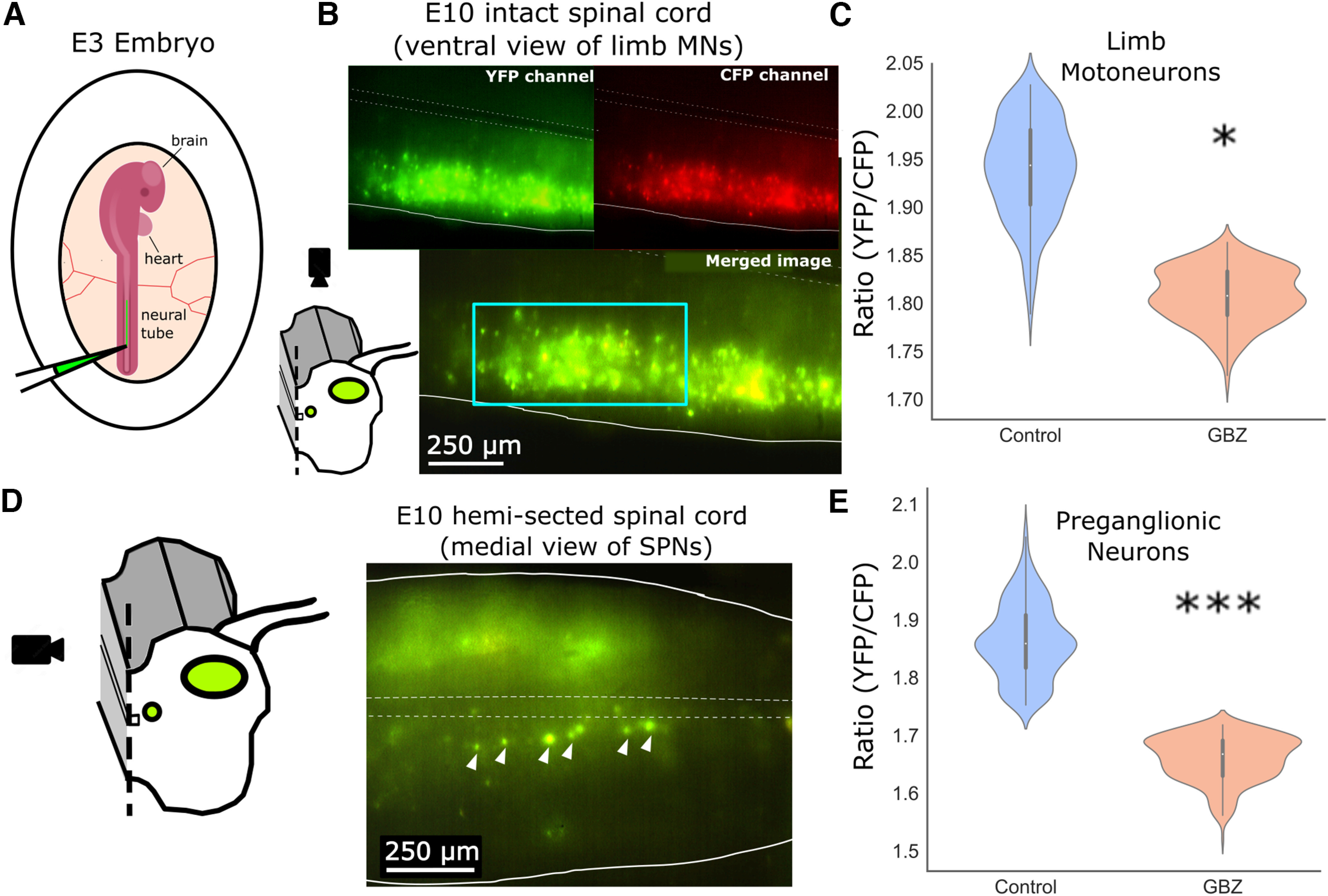
Intracellular chloride levels of MNs and SPNs were altered after synaptic blockade. ***A***, Clomeleon electroporation into the neural tube at embryonic day 3 (E3). ***B***, YFP, CFP, and merged image of ventral view of spinal cord were used to analyze chloride levels in somatic MNs in E10 spinal cord. Schematic at bottom left shows orientation of ventral view. ***C***, Analysis of MNs showed significant reduction in YFP/CFP ratio in drug-treated group. In order to examine entire dataset while controlling for unbalanced number of cells observed between cords, hierarchical bootstrapping was implemented. This analysis of resampled data revealed lower ratios in GBZ-treated cells (1.79 ± 0.03) compared with vehicle (1.92 ± 0.05, *p* = 0.035,*). ***D***, Left, Medial view of hemisected spinal cord was imaged for analysis of SPNs. Right, Merged YFP-CFP image, with SPNs designated with white arrow, at E10 in embryos which were labeled with Clomeleon at E3. ***E***, Analysis of SPNs also revealed a reduction in ratio in GBZ-treated cells. Hierarchical bootstrapping test confirmed lower ratios in GBZ-treated cords compared with control cords (GBZ =1.66 ± 0.04, H_2_O = 1.86 ± 0.07, *p* < 0.0001,***). Finally, linear mixed effects test was performed on entire dataset, which determined difference between groups is explained by fixed effect of treatment group, not driven by random effects such as embryo number or experiment date (*p* < 0.005). See Extended Data Figure 2-1 for additional statistical treatment of data.

To examine whether intracellular chloride levels are increased in SPNs after 48-h GABAergic blockade, as would be expected for GABAergic scaling, embryos were electroporated at E3 with plasmids containing the Clomeleon gene, a fluorescence resonance energy transfer (FRET)-based, ratio-metric chloride indicator (Fig. 2*A*). Embryos were dissected at E10, and cords were imaged thereafter. We first examined the pattern of expression in the somatic MNs to determine the specificity of the label. As described previously (Lindsly et al., 2014), Clomeleon expression was typically in the lateral half of the hemicord when imaging through the ventral surface of the cord, where somatic MNs are located (Fig. 2*B*). This pattern of expression in somatic MNs is likely a result of electroporating the plasmids at a developmental stage in which these cells undergo their final mitotic division (Hollyday and Hamburger, 1977) thus preventing further dilution of the genetic indicator that would occur with additional divisions. Cords were therefore only accepted into the dataset if they displayed this distinct lateral hemicord expression pattern (Fig. 2*B*). We imaged somatic motoneurons through the ventral white matter (Fig. 2*B*), as we had done previously (Lindsly et al., 2014). We found that ratios were significantly reduced following *in ovo* 48 h treatment with the GABA_A_R antagonist gabazine (10 μm) from E8 to E10, compared with ratios from vehicle-treated embryos (Fig. 2*C*; Extended Data Fig. 2-1). This difference in FRET ratios of YFP:CFP in the somatic MNs was consistent with our prior study in these cells (Lindsly et al., 2014) and matched the chloride concentrations that were previously determined using whole cell and perforated patch techniques, thus demonstrating an increase in intracellular chloride concentration associated with GABAergic synaptic scaling (Gonzalez-Islas et al., 2010). Because SPNs are born at the same time as somatic MNs they should also be labeled with Clomeleon (Stifani, 2014). To confirm this, we looked for Clomeleon-labeled cells viewed from the medial surface of the hemisected spinal cords (Fig. 2*D*) that were in the previously identified location of the SPNs (Fig. 1*C*). Indeed, we identified many medially positioned Clomeleon-expressing cells in the SPN location (Fig. 2*D*). These cells were then analyzed for Clomeleon ratios to identify differences in chloride levels between treatment groups. Using multiple analyses of FRET ratios, we found significant reductions in YFP:CFP ratios of embryos treated with gabazine for 48 h compared with those treated with vehicle (Fig. 2*E*; Extended Data Fig. 2-1). This was consistent with our previous work on somatic MNs (Lindsly et al., 2014) and suggested that chloride levels were elevated in response to GABAergic blockade as there is an inverse relationship between the ratio of YFP:CFP and the intracellular chloride concentration. While this result demonstrates that intracellular chloride levels have increased in SPNs, we cannot provide a precise change in concentration because we did not redo the necessary calibration experiments on these more superficially located SPNs.

10.1523/ENEURO.0297-23.2023.f2-1 Extended Data Figure 2-1 Statistical confirmation of Clomeleon data. ***A***, Motoneuron dataset showed significant difference between control and drug-treated groups. An independent, two sample *t* test was performed, restricting data to 10 cells per cord to handle uneven sampling between cords. This test revealed that ratio of YFP:CFP intensity was lower in cells from embryos treated with GBZ (1.81 ± 0.01) than from those treated with vehicle (H_2_O, 1.94 ± 0.02, *t*_(175)_ = 6.7, *p* < 0.001). ***B***, Full dataset is shown here, including every cell from each cord. This dataset is not suitable for a *t* test, however linear mixed effects modeling revealed a significant difference between groups was driven primarily by treatment group, not driven by cord umber or experiment date (*p* < 0.001). ***C***, Analysis of SPNs also revealed a reduction in ratio in treated cells. An independent, two sample *t* test using four cells per cord revealed that FRET ratio of YFP:CFP were significantly lower in spinal cords from embryos treated with GBZ (1.65 ± 0.03) compared to those treated with H_2_O (1.86 ± 0.02, *t*_(49)_ = 5.9, *p* < 0.001). ***D***, Full dataset is shown here. As number of cells was imbalanced between cords, data was unsuitable for *t* test. Therefore, linear mixed effects test was performed on entire dataset. This test determined a significant difference between groups that was explained by treatment group alone, thus not driven the random factors of cord number or experiment date (*p* < 0.005). Bars represent standard error. Notches in bars represent mean. Download Figure 2-1, TIF file.

In summary, lumbosacral SPNs, as defined in methods, exhibit an increase in intracellular chloride after 48 h of synaptic blockade of GABA_A_ receptors. This suggested that GABAergic synaptic scaling occurred in both SPNs and somatic MNs, indicating the presence of synaptic HP in the central arm of the sympathetic nervous system during embryonic development.

### Cellular excitability decreased in PGNs following 2-d synaptic blockade *in ovo*

#### Development of synaptic activity

In order to further characterize the capacity for HP in the embryonic sympathetic nervous system, we tested the downstream synaptic target of the central SPNs, the PGNs of the peripheral nervous system. This allowed us to test whether PGNs themselves might compensate for plasticity occurring in the SPNs or act as a simple relay. As PGNs receive exclusively cholinergic input from SPNs, we attempted to trigger HP with *in vivo* pharmacological block of ionotropic nicotinic receptors, using hexamethonium (Hex), a ganglionic nicotinic acetylcholine receptor antagonist.

**Figure 3.**
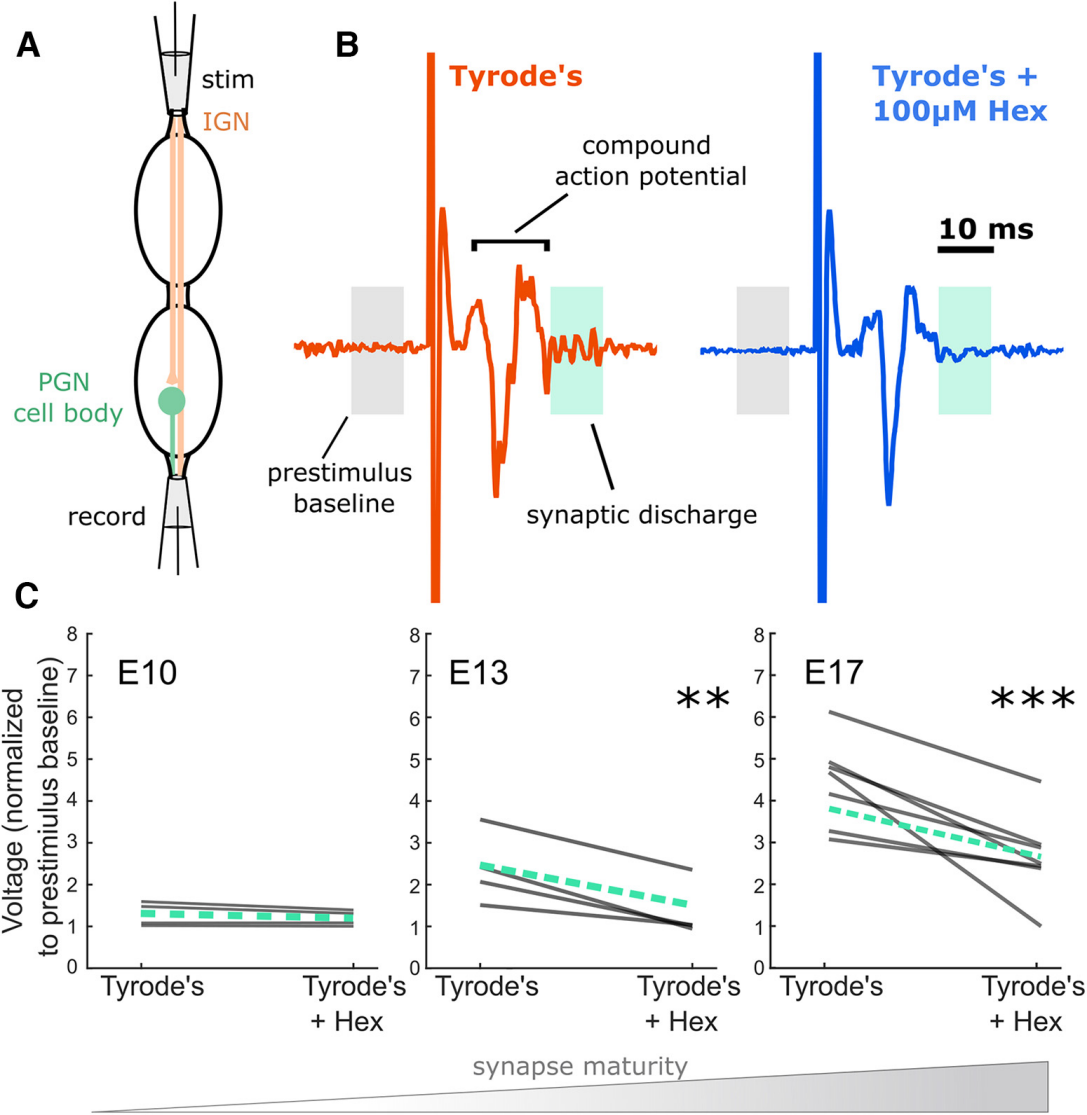
PGNs develop mature synaptic activity between E13 and E17. ***A***, Schematic of PGN extracellular recording set up. ***B***, Example traces at E17 show evoked response, including synaptically-driven discharge in PGNs (highlighted in green box, 10 ms), followed by a reduction in discharge after the addition of 100 μm Hex into the bath. ***C***, The reduction of discharge was calculated across many trials for the following developmental stages: E10, E13, and E17 (gray lines represent individual experiments, green dashed line represents mean). Paired, two tailed *t* tests revealed the following: at E10, there was no difference in discharge after the addition of Hex (*t*_(3)_ = 1.8, *p* = 0.17). At E13, there was a significant reduction in synaptic discharge with Hex (*t*_(3)_ = 6.6, *p* < 0.01, **). At the E17 stage, the activity was significantly reduced after addition of 100 μm Hex (*t*_(6)_ = −4.6, *p* < 0.005, ***).

We first sought to better define the developmental stage at which the SPN-to-PGN connections were established (Hruschak et al., 1982; Dryer and Chiappinelli, 1985; Moss and Role, 1993; Devay et al., 1994) and to ensure the synaptic connections were strong enough to reliably induce spiking activity in the postsynaptic PGNs. To this end, we isolated several segments of the paravertebral chain between the second lumbosacral segment (LS2) and LS7 and stimulated the SPN axons using a suction electrode containing the IGN between LS1 and LS2 (Fig. 3*A*). Therefore, stimulating the rostral end of the tissue allowed us to stimulate any preganglionic axons which were projecting in the caudal direction from LS1 and above (Yip et al., 1998), while recording the resulting volley through preganglionic fibers as well as synaptically-driven spiking activity in the postsynaptic PGNs. The recording suction electrodes were attached to the IGN at LS5, LS6, or LS7. Stimulations were provided every 60 s, at 4× threshold for eliciting the compound action potential volley (Fig. 3*B*). The PGN discharge was measured as the average of the rectified, filtered voltage trace for the indicated 10 ms period (Fig. 3*B*, green box) normalized to the 10-ms prestimulus period (Fig. 3*B*, gray box). To confirm that activity observed was induced by synaptic transmission, we added the nicotinic receptor antagonist Hex (100 μm) to the circulating solution (Fig. 3). At E10, minimal synaptic activity was observed, but did not surpass 2× baseline noise levels (Fig. 3*C*, green, dashed line represents mean). Further, the addition of 100 μm Hex did not significantly affect these measures of synaptic discharge in any of the E10 experiments (Fig. 3*C*). At E13, three out of four preparations exhibited synaptic discharge greater than 2× that of the baseline, which was significantly lower with the addition of 100 μm Hex (Fig. 3*C*). At E17, all seven preparations exhibited clear synaptic discharge, larger than that which was observed at E13 (between 3.1× and 6.2× baseline amplitude; Fig. 3*C*). This prominent synaptic activity at E17 was significantly reduced by addition of 100 μm Hex (Fig. 3*C*). Importantly, preparations that did not express synaptic discharge were only included in the analysis if the stimulus reliably produced a clear orthodromic volley, demonstrating that the presynaptic SPN projections were successfully stimulated and projected down to that ganglion. Taken together, this evidence demonstrated that E13 is the developmental age at which the impact of synaptic transmission begins to increase rapidly. Therefore, to test for a homeostatic response we chose to begin synaptic blockade at this time point.

#### Intrinsic excitability of PGNs

Measuring scaling in PGNs was not possible, as the typical method for determining scaling involves measuring the amplitude of mPSCs, but these spontaneous events were infrequent in this preparation. Instead, we examined homeostatic changes in intrinsic excitability, as this mechanism of HP has been observed in embryonic spinal MNs following synaptic blockade (Wilhelm et al., 2009).

**Figure 4.**
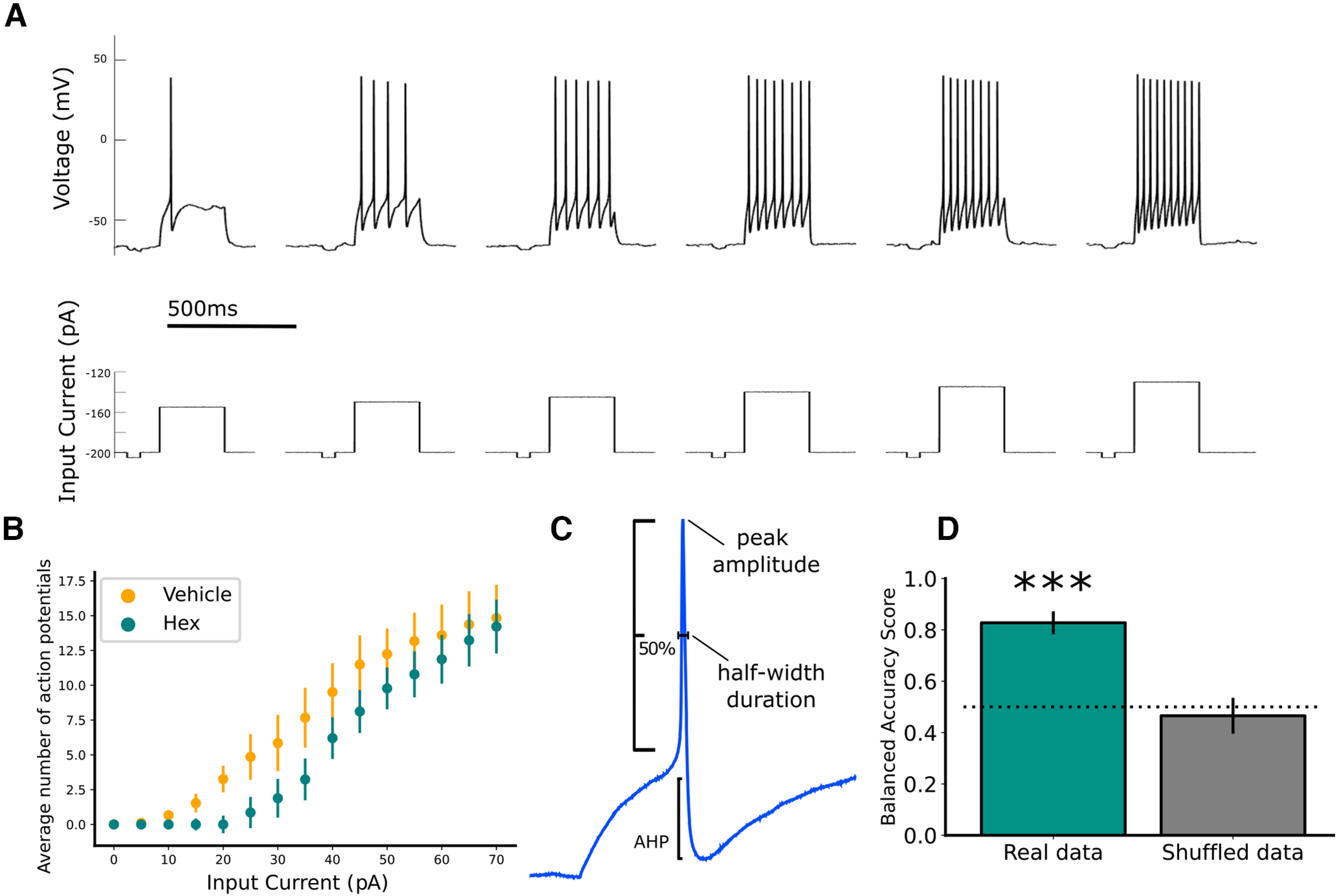
Nicotinic blockade led to a change in excitability in PGNs that did not appear to be in a compensatory direction. ***A***, Progressively increasing depolarizing current steps (500 ms in duration, intervals of 5 pA) were delivered every other second. ***B***, Frequency/current (*f*/I) curve reveals the relationship between treatment condition and excitability of cells. Two-way repeated measures ANOVA revealed a main effect of input current on output number of action potentials, as well as a significant between-group effect of treatment (*F*_(14)_ = 32.8, *p* < 0.0001). Because cords were not evenly sampled, and because of the hierarchical nature of the dataset, a linear mixed effect (LME) model was performed on a linear portion of the dataset (20 to 55 pA) to verify this effect of treatment group. This analysis showed a significant effect of treatment group (*t*_(15.49)_ = −2.28, *p* = 0.037), and eliminated any effect of potential confounding factors such as experiment date. ***C***, Schematic representation of parameters measured from individual spikes, summarized in Table 1. ***D***, To confirm the effect using all input current values, logistic regression model was trained on a subset of data to predict the treatment group. When given naive data from the test dataset, the mean accuracy of the model was significantly greater than chance (mean accuracy = 82.7%, *t*_(28)_ = 7.29, *p* < 0.001, ***). In comparison, the model’s accuracy when predicting treatment group for a shuffled dataset, was no better than chance (mean accuracy = 46.6%, not different from chance accuracy of 0.50 (*t*_(28)_ = 0.63, *p* = 0.54), further validating the effect of treatment group. Error bars represent standard error.

Because the SPN to PGN synaptic connection was shown to be functionally intact and able to produce reproducible synaptic discharge beginning at E13, this developmental stage was used to block synaptic input to PGNs. We assessed homeostatic changes in intrinsic excitability 48 h later, at E15. At this time, the sympathetic chain was isolated, and cellular excitability was examined via whole cell recordings of PGNs. Values were compared between embryos treated for 48 h with 100 μm Hex and those treated with vehicle (dH_2_O). Neurons had a mean resting potential of −44.5 mV and were held in current clamp at −70 ± 5 mV, (without adjusting for liquid junction potential). Progressive depolarizing steps (500-ms duration, 5-pA step interval) were given (Fig. 4*A*). Surprisingly, following 2-d *in ovo* blockade of ganglionic nicotinic transmission, *f*/I relationships from Hex-treated embryos showed a rightward shift compared with those from vehicle-treated embryos, demonstrating that intrinsic excitability in PGNs had been reduced after nicotinic blockade (Fig. 4*B*). To validate this finding with more rigorous tests, linear mixed effects and logistic regression for sigmoidal curves were used, confirming the strong effect of Hex treatment in reducing intrinsic excitability (Fig. 4*D*). This result was opposite to what we would expect if there had been a compensatory shift toward increased excitability in response to the reduction of excitatory synaptic drive. To better understand the effect of Hex treatment on PGNs, we performed *post hoc* analysis of membrane properties and these values are summarized in Table 1. Peak amplitude of action potentials was significantly higher in cells from embryos that were treated with Hex compared with those treated with vehicle (Table 1). The half-width duration was also 38% shorter, on average, in the hex-treated condition. However, this measure fell just outside of statistical significance (*p* = 0.051). It is important to recognize that our recordings likely captured a diversity of ganglionic neuron subtypes (Ernsberger et al., 2020), but the present study was not able to differentiate subpopulations of PGNs. Taken together, these differences suggest that while there may have been some adaptation in various ion channels, there was no homeostatic shift in overall intrinsic excitability.

#### No homeostatic adjustment to synaptically-evoked PGN discharge following acute nicotinic blockade

**Figure 5.**
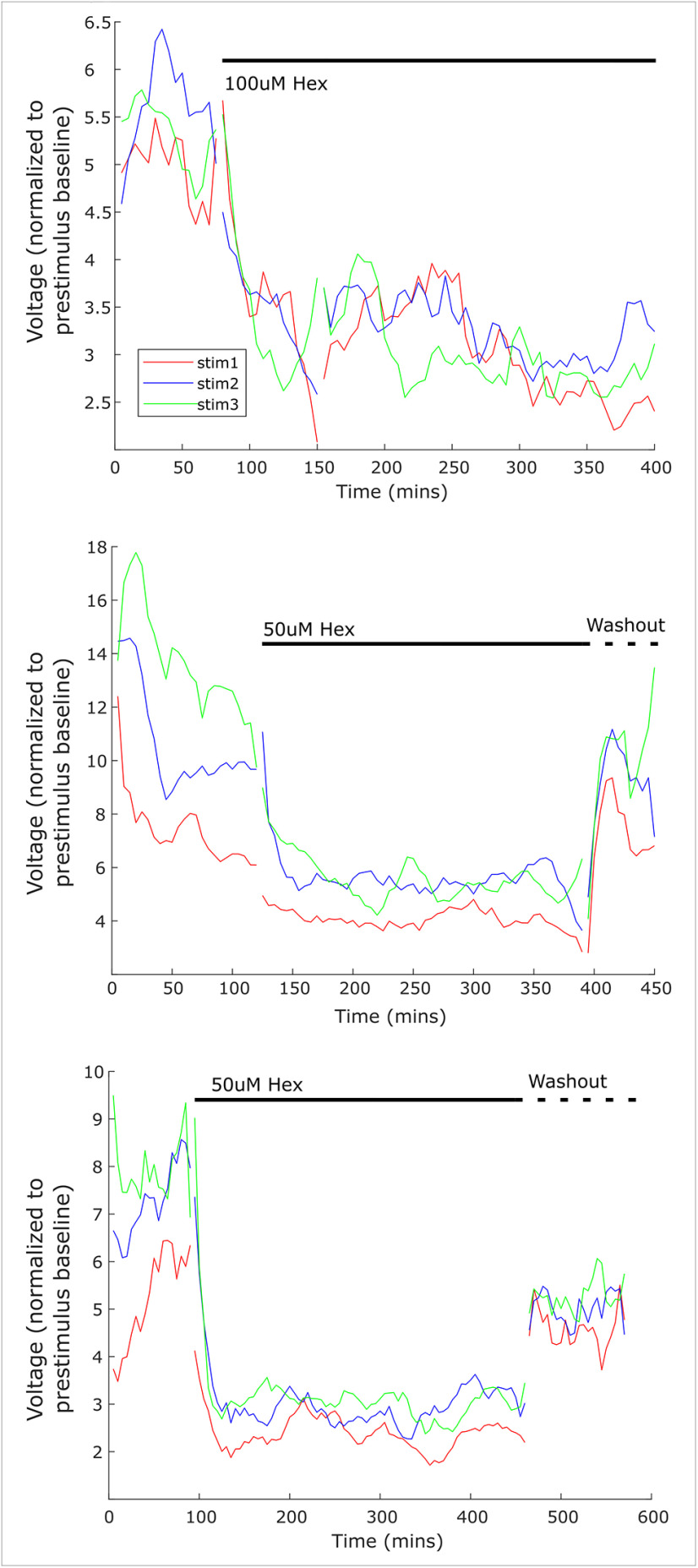
Acute nicotinic blockade resulted in reduction of synaptic activity which did not recover over several hours. *Ex vivo* preparations of sympathetic chain tissue were used for extracellular recordings in baseline conditions, and during nicotinic blockade using either 50 or 100 μm hexamethonium (Hex). Synaptic activity was evoked using a train of three stimulations (20-Hz interval) every 5 min, and the resulting synaptic discharge was calculated at each time point. The synaptic discharge remained reduced for several hours, such that baseline levels of synaptic activity did not recover for 4–5 h of nicotinic blockade. In two experiments, washout of the drug was conducted. Synaptic activity returned after washout, demonstrating that the tissue was still healthy.

This decrease in membrane excitability was in the opposite direction than expected for the expression of HP. However, there are some forms of homeostatic synaptic plasticity that occur rapidly, including acute changes in intrinsic excitability that take place within hours of blockade (Gonzalez-Islas et al., 2020). To determine whether we could observe an acute homeostatic adjustment to an evoked response, we reduced synaptic input while examining the SPN-evoked response in the sympathetic chain *ex vivo* over several hours. Here, we used E17 preparations which express robust evoked firing, such that it would be possible to observe discharge even after adding Hex (Fig. 5; also see Materials and Methods, Extracellular electrophysiology). We stimulated SPNs with a brief train of three pulses (20 Hz) every 5 min, comparing synaptic discharge before and after adding sub-saturating concentrations (50–100 μm) of Hex to the bath. We found that this reduction of synaptic input to PGNs led to a reduction of measured synaptic activity, which showed no evidence of recovering to baseline amplitude levels through the observed 4-h drug application period (Fig. 5). Importantly, in the two experiments where a washout was conducted, activity recovered toward baseline levels, indicating that the lack of recovery during the Hex condition was not because of the deterioration of the tissue.

## Discussion

The autonomic nervous system homeostatically maintains certain features of body function, such as blood pressure. This system adjusts its output within seconds to accommodate challenges to baseline blood pressure. When challenges are no longer present, the output of the autonomic nervous system returns to baseline. It is unknown how this baseline autonomic output is established during development. Spinal and peripheral neurons of the sympathetic nervous system were examined for evidence of HP during embryonic development. In the central arm of the sympathetic nervous system, SPNs were examined for evidence of GABAergic synaptic scaling. An increase in intracellular chloride concentration was found in the cell bodies of the SPNs 48 h after *in ovo* GABA_A_ receptor blockade. This suggests that GABAergic scaling has occurred in SPNs and demonstrates that the mechanism is through chloride accumulation (Gonzalez-Islas et al., 2010). In the peripheral arm, however, the PGNs did not appear to exhibit homeostatic increases in overall intrinsic membrane excitability. After 48 h of nicotinic synaptic block, PGNs were less excitable compared with those from vehicle-treated embryos. In separate *ex vivo* experiments at E17, SPN-evoked discharge in PGNs was reduced by acutely inhibiting synaptic transmission, which did not show any signs of recovering within 4 h of antagonist application (Fig. 5). Responses to synaptic perturbations of both SPNs and PGNs could contribute to the critical period for the establishment of baseline autonomic activity.

Following 48-h, *in ovo* blockade of GABA_A_ receptors at early developmental stages when GABA is known to be excitatory in several circuits (Ben-Ari, 2002), intracellular chloride concentrations of SPNs were increased compared with control cords. This indicated that chloride-mediated synaptic scaling had occurred. The finding recapitulated previous studies in somatic MNs of the spinal cord at the same stages, in which the chloride concentrations increased following 24 or 48 h, but not 12 h, of GABA_A_R blockade. In addition, these previous studies in MNs directly demonstrated increases in mPSC amplitude, driven by a depolarization of the chloride reversal potential as demonstrated by both perforated patch and whole cell recordings of mPSCs (Gonzalez-Islas et al., 2010; Lindsly et al., 2014). These results are the first that we know of to suggest that GABAergic synaptic scaling, or any form of HP, is expressed in developing sympathetic spinal neurons. We have also demonstrated that these SPNs, like MNs, are among the spinal circuitry that participates in SNA (Fig. 1*D*), a phenomenon which has been shown to be important for proper motor development (O’Donovan et al., 1998) and maturational tuning of spinal cord synaptic strength (Gonzalez-Islas and Wenner, 2006). Because SPNs are autonomic MNs that arise from the same progenitor population as somatic MNs (Stifani, 2014) it may not be surprising that both SPNs and MNs express SNA and this form of synaptic scaling. In addition, this phenomenon of postsynaptic scaling has been observed in MNs as well as spinal interneurons (Lindsly et al., 2014), indicating that this form of HP may be part of a universal mechanism for regulating synaptic strength in the ventral horn of the spinal cord during early development. It is possible that the SPNs exhibit other HP mechanisms, such as those expressed in MNs, but more tests will be necessary to determine this going forward.

The finding that blockade of GABAergic transmission triggers an increase in GABAergic synaptic strength in somatic and sympathetic MNs raises the question: what is the homeostatic goal of GABAergic scaling in this system? While we have not directly addressed this question in the current study, it is clear that the predominant role of GABAergic scaling in somatic MNs of the embryonic spinal cord is the maintenance of GABAergic synaptic input (Wenner and Pekala, 2022). Our previous work in somatic MNs has shown that GABAergic scaling (by intracellular chloride regulation) is triggered by changes in GABA_A_ receptor activation rather than changes in spiking activity (Garcia-Bereguiain et al., 2016). If reductions in GABAergic transmission occur during a defined critical period, then increases in intracellular chloride levels could be triggered. If the perturbation is maintained beyond the closing of the critical period, then this could leave the sympathetic circuitry in a hyperexcitable state. This could underlie the vulnerability to perturbations in autonomic signaling during early developmental time points described above (Mulkey and du Plessis, 2019). This is the first demonstration we are aware of that shows HP in developing SPNs, which could contribute to a critical period for the maturation of sympathetic circuit excitability. An important next step would be to determine whether this or other homeostatic mechanisms are expressed during a limited developmental window that could alter sympathetic tone over the long-term.

On the other hand, PGNs in the peripheral arm of the sympathetic nervous system did not show evidence of compensatory increases in overall intrinsic excitability following synaptic input blockade (Figs. 4, 5). In fact, the difference in *f*/I curves suggests a reduction in excitability after a 2-d blockade of nicotinic synaptic input. This is consistent with a previous study that demonstrated that sympathetic drive was reduced following *in vivo* silencing of the superior cervical ganglion-pineal circuit (Calinescu et al., 2011). In addition, membrane properties were compared between treatment groups. Only AP peak amplitude significantly differed between treatment groups (Table 1), which could suggest a change in voltage-gated Na^+^ channel currents. However, any such adjustment did not ultimately result in an overall increase in excitability 48 h after synaptic blockade with Hex. Moreover, when we examined the PGN circuit *ex vivo* at E17, no recovery of synaptically-induced activity was detected during a 4-h application of sub-saturating ganglionic nicotinic antagonist. This suggests that compensatory plasticity is not present on this acute timescale at this age (Fig. 5). This finding also argues against the expression of rapid forms of HP such as presynaptic HP (Davis, 2006; Goel and Dickman, 2021; Engisch et al., 2022). Together, our measures of excitability and input/output function of the PGNs after synaptic blockade did not support the expression of HP during embryonic development. In this case, it may be that PGNs do not express compensatory plasticity, instead acting as a simplistic relay to the target tissue at this stage, as some groups suggest (McLachlan, 2003). Alternatively, we cannot rule out the possibility that the PGNs express a different form of homeostatic response that was not detected, or that was expressed at a different developmental stage. At E13 and even at E17, PGNs are still relatively immature as several synaptic markers continue to develop well into the posthatching period (Black et al., 1971; Greene, 1976; Hruschak et al., 1982). Therefore, the critical period for the expression of HP may exist at a later time point for PGNs.

Indeed, while previous work on this topic is limited, some studies suggest that PGNs have a homeostatic capacity in the adult system (Wang et al., 2010; Djemil et al., 2023). Evidence for presynaptic HP has been shown in adult thoracic PGNs in response to an antibody-mediated perturbation of their synaptic input (Wang et al., 2010). The neuromuscular junction (NMJ) expresses this form of presynaptic HP in multiple species (Goel and Dickman, 2021; Engisch et al., 2022), and shares similarities with the SPN to PGN synaptic connection. However, we saw no evidence of such presynaptic HP in our work (Fig. 5). Further investigation is needed to determine whether SPN scaling or reduced PGN excitability play a role in establishing the sympathetic tone and whether these mechanisms could contribute to the development of sympathetic disorders later in maturation (Crump et al., 2011; Mulkey and du Plessis, 2019).

The finding that one sympathetic cell type (SPN) expressed a form of HP in response to 48-h synaptic blockade, while another (PGN) did not, could suggest that during embryonic development, the central and peripheral components of the sympathetic nervous system each play a different role in the overall maintenance of autonomic setpoint. This discrepancy could be because of intrinsic molecular differences in these cell types. In addition, SPNs and PGNs are at different relative stages of developmental maturity, which may impact their capacity for engaging in homeostatic adjustments. For example, the SPNs undergo their final cell division by E5 and have migrated to their mature location near the central canal to form the column of Terni by E8 (Levi‐Montalcini, 1950; Prasad and Hollyday, 1991). While it is clear that the SPNs are synaptically-driven to express SNA at E10 (Fig. 1*D*) and potentially by E4 when somatic MNs express SNA (Milner and Landmesser, 1999), the PGNs do not appear to be synaptically activated until ∼E12 (Fig. 5; also Dryer and Chiappinelli, 1985; Moss and Role, 1993). Thus, the E13 stage at which PGN input was blocked, is a stage of relative immaturity when compared with the SPNs, and therefore may not have captured their capacity for HP that could exist at later developmental stages. We have demonstrated HP in the developing sympathetic nervous system, and an important next step will be to determine whether this plays a longer-term role in autonomic dysfunction following an early life perturbation.

## Synthesis

Reviewing Editor: Matthew Grubb, King’s College London Decisions are customarily a result of the Reviewing Editor and the peer reviewers coming together and discussing their recommendations until a consensus is reached. When revisions are invited, a fact-based synthesis statement explaining their decision and outlining what is needed to prepare a revision will be listed below. The following reviewer(s) agreed to reveal their identity: John Horn. Note: If this manuscript was transferred from JNeurosci and a decision was made to accept the manuscript without peer review, a brief statement to this effect will instead be what is listed below.

Synthesis:

Both Reviewers are extremely positive about the manuscript. They both make many suggestions for improvement, and all of their issues should be addressed in a revised version. Please note that responding to Reviewer 2’s points #1 (definition of homeostasis and its relevance to the findings) and #3 (critical period) does not necessitate new data or analysis. Instead, please add a thorough treatment of each question in the revised Discussion. As the Reviewer stated in our post-review consultation: ‘“Homeostasis” used as a noun implies that some measurable, physiological parameter is being maintained in order to support a behavior or a function. For these neurons, I think it would be fine for the authors to speculate in the Discussion on what that measure is (firing rate?)’ & ‘I just wanted more of an explanation about why homeostasis would be playing a special role to dictate an early critical period.’ I look forward to reading a revised re-submission with the appropriate changes.

Reviewer 1:

This well-executed and interesting study examines whether homeostatic mechanisms regulate the excitability of sympathetic preganglionic and postganglionic neurons using chick embryos as the model system. Previous work from other labs has shown that in many neuronal networks, ‘homeostatic’ mechanisms maintain levels of activity by adjusting synaptic weights and postsynaptic excitability. Authors of the current study have extended this idea to spinal somatic motorneurons by showing that blocking GABA-ergic inhibition, raises intracellular chloride concentrations and shifts the chloride equilibrium potential in the depolarizing direction. Importantly GABA-ergic activation of chloride channels has an excitatory action during embryonic development. Building upon the earlier work, the current study asks whether similar homeostatic mechanisms of plasticity operate in embryonic sympathetic spinal preganglionic neurons and in ganglionic neurons. Improved understanding of early autonomic development has important implications for normal autonomic function and for medical syndromes arising from premature birth and other developmental disorders.

This manuscript has numerous strengths.

1) The text is clearly written and should be readily understood by specialists and non-specialists alike.

2) The model system is appropriate for the questions being asked.

3) The methods for localizing preganglionic neurons and somatic neurons are carefully executed. The methods for calcium and chloride imaging, for extracellular and patch recording from sympathetic postganglionic neurons are appropriate.

4) The statistical methods are appropriate. They account for the nested design of key experiments and use appropriate tests.

5) The conclusions are well justified and set the stage for future work. Importantly the authors refrain from overinterpreting their data.

Several minor corrections or changes are recommended.

Line 85- the methods state that E10-17 embryos were used. However, electroporation was done as early as E3 (e.g. line 131, line 226 and line 246).

Line 262-3 A reference should be added concerning the birthdates of SPNs and MNs. It is in the discussion (Stifani 2014, line 383), but would be helpful here as well.

Lines 297 -299, “At E13, 4 out of 5 preparations....” In Fig. 3B, it looks like 5 of 6 preparations. “At E17, all 7 preparations...” In Fig 3 B it looks like 9 preparations.

Fig 3B- add calibration bars to the physiological recordings.

Table 1 should have a brief legend. Are the data expressed as mean {plus minus} sd or sem? Presumably the parenthetic numbers with t-stats reflect the n’s in two-tailed unpaired t-tests?

Line 402 -The authors interpret the increased AP amplitudes in table 1 as evidence for increased sodium currents. If so, then one would also expect an increase in dv/dt for the upstroke. However, the upstroke velocity was unchanged.

Reviewer 2:

In this manuscript, the authors use the chick embryo sympathetic nervous system (SNS) to examine whether sympathetic preganglionic neurons (SPNs) exhibit a novel form of homeostatic plasticity.

The data in the study support a homeostatic mechanism at the GABAergic synapses of SPNs. When the developing animals (embryonic) are exposed to gabazine to block the GABAA receptors, there is evidence that intracellular SPN chloride concentration increases. At this early stage of development, GABAA receptors are excitatory because the chloride gradient is reversed (low outside and high inside, early in development). Therefore, an increased intracellular chloride concentration would support excitatory activity as an offsetting response to blockade of excitatory GABAA receptors. The authors term this response “GABAergic synaptic scaling” and draw parallels to homeostatic mechanisms they documented in somatic motor neurons.

The authors then extend their idea to test whether homeostatic mechanisms are expressed in the synaptic targets of SPNs, the postganglionic neurons (PGNs). However, in vivo or acute blockade of the PGN nicotinic acetylcholine receptors do not induce discernible homeostatic adjustments in PGN excitability. In fact, in some ways, the adjustments seem to be Hebbian (less excitability).

Overall, this is an exciting study because it uncovers a potentially novel form of homeostatic plasticity in the chick embryo. The authors speculate that such a form of homeostasis could play a role in the early establishment of a setpoint of excitability, which is an intriguing explanation for the plasticity. Several areas in the manuscript can be improved to strengthen specific ideas and expand our understanding of plasticity in preganglionic and postganglionic neurons of the sympathetic nervous system during embryonic development. Major and minor points are listed below.

Comment to the authors.

Major points:

1. Increased chloride concentration (and thus increased the driving force for depolarizing currents) is just a single measure. It may be somewhat challenging for readers to conclude for certain that this is a homeostatic adjustment in the absence of some other independent measure that demonstrates a tangible offsetting effect. Do the authors have an idea about what physiological parameter is being maintained for the SPNs (other work speculates that GABAergic scaling might support firing rate homeostasis)? Or is there some sort of independent physiological measure that would demonstrate that the increased intracellular chloride is having a discernible, offsetting effect?

2. (Related to 1): Do the authors have a sense of how the Clomeleon YFP/CFP ratios in this study (Fig. 2) translate to a specific measure of intracellular [Cl-]? The question is whether this change would be of a large enough magnitude to matter for excitability. In the Lindsly et al. 2014 paper (Fig. 3 of that study), there is a graph that estimates [Cl-] for a given ratio. If that graph were to apply to the ratios in this study, then the changes in chloride concentration would be in a narrow range (rising from ∼60 mM to ∼80 mM).

3. The idea of a stage-specific critical period is intriguing, but as written, it is a vague idea. It might be necessary to expound on this because - if this reviewer is interpreting correctly - the early timepoint observation is the main advance of this study beyond the similar observations in prior papers (e.g., Lidsly et al.).

So, beyond timing of when the authors looked, is there a reason to think that this form of plasticity contributes to an “early critical period” (Line 274)? This reviewer wonders why stage-specific homeostasis would be beneficial and how would a mode of synaptic scaling specifically contribute to an early critical period of ANS development - as opposed to being present throughout life to keep activity steady? The authors could expound on this in the Discussion section.

4. (Related to 3) Other studies examining homeostatic plasticity have shown that it can be subdivided into rapid or chronic forms in the nervous system. Do the authors have speculation on the timescale (and perdurance) of this form of homeostasis? This could also be highlighted in the Discussion.

Minor points:

1. The author should clearly explain the conditions they refer to GABAergic as excitatory, simply because the idea of GABAergic signaling being excitatory will be a little counterintuitive to most readers.

2. This manuscript contains a lot of field-specific abbreviations that share letters or terminology with one another (SPN, ANS, SNS, etc.). A reader not in the immediate field might have difficulty with those abbreviations.

3. In the first section, the authors discuss chloride-mediated signaling in SPNs, where GABA is vital in mediating homeostasis. In the second part, there is a sudden shift to the cholinergic inputs from SPNs to PGNs. It is not clear to this reader exactly why one would necessarily predict (a priori) that the PGNs would execute a similar form of plasticity as the SPNs. Is it because they are both excitatory and in a linear circuit? What does it mean for the system that the PGNs seem not to show a homeostatic response?

4. Why would a shortened AP half-width predict activity in a compensatory direction? Quite the opposite, it might imply a briefer pulse for neurotransmission.

## Author Response

We thank the editor and reviewers for their comments and have addressed them individually below (blue text) and in the text of the manuscript (green) and hope this satisfies all concerns.

Synthesis:

Both Reviewers are extremely positive about the manuscript. They both make many suggestions for improvement, and all of their issues should be addressed in a revised version. Please note that responding to Reviewer 2’s points #1 (definition of homeostasis and its relevance to the findings) and #3 (critical period) does not necessitate new data or analysis. Instead, please add a thorough treatment of each question in the revised Discussion. As the Reviewer stated in our post-review consultation: ‘“Homeostasis” used as a noun implies that some measurable, physiological parameter is being maintained in order to support a behavior or a function. For these neurons, I think it would be fine for the authors to speculate in the Discussion on what that measure is (firing rate?)’ & ‘I just wanted more of an explanation about why homeostasis would be playing a special role to dictate an early critical period.’ I look forward to reading a revised re-submission with the appropriate changes.

Reviewer 1:

We thank the reviewer for their very positive words about our manuscript and respond to their minor comments below.

Line 85- the methods state that E10-17 embryos were used. However, electroporation was done as early as E3 (e.g. line 131, line 226 and line 246).

Electroporation was carried out at E3 and and we have corrected this (now lines 88 & 134).

Line 262-3 A reference should be added concerning the birthdates of SPNs and MNs. It is in the discussion (Stifani 2014, line 383), but would be helpful here as well.

This reference has been added (on what is now line 274).

Lines 297 -299, “At E13, 4 out of 5 preparations....” In Fig. 3B, it looks like 5 of 6 preparations. “At E17, all 7 preparations...” In Fig 3 B it looks like 9 preparations.

The dashed green line in Figure 3 is the ‘average’ line for all preparations at a given age. We have now made this clearer in the figure legend, and in the associated text description (line 314).

Fig 3B- add calibration bars to the physiological recordings.

This was addressed in the final figure.

Table 1 should have a brief legend. Are the data expressed as mean (plus minus) sd or sem? Presumably the parenthetic numbers with t-stats reflect the n’s in two-tailed unpaired t-tests?

We have added a legend which includes a description of the statistical test.

Line 402 -The authors interpret the increased AP amplitudes in table 1 as evidence for increased sodium currents. If so, then one would also expect an increase in dv/dt for the upstroke. However, the upstroke velocity was unchanged.

We agree with the reviewer and a similar comment of the second reviewer. Because of this we have removed or softened any statements about altered currents based on AP waveform (line 431).

Reviewer 2:

We thank the reviewer for their comments and respond to their major and minor points below.

Major points:

1. Increased chloride concentration (and thus increased the driving force for depolarizing currents) is just a single measure. It may be somewhat challenging for readers to conclude for certain that this is a homeostatic adjustment in the absence of some other independent measure that demonstrates a tangible offsetting effect. Do the authors have an idea about what physiological parameter is being maintained for the SPNs (other work speculates that GABAergic scaling might support firing rate homeostasis)? Or is there some sort of independent physiological measure that would demonstrate that the increased intracellular chloride is having a discernible, offsetting effect?

Based on our previous work, we believe that the predominant role of GABAergic scaling in the embryonic spinal cord is the maintenance of GABAergic synaptic input, although obviously this could influence network activity. Our previous work (Garcia-Bereguiain et al 2016) has shown that GABAergic scaling (by intracellular chloride regulation) is triggered by changes in GABAA receptor activation rather than changes in spiking activity. Based on this and other findings, we believe that GABAergic scaling in SPNs, like MNs, is sensing and attempting to regulate synaptic strength in a compensatory manner as we described previously (Garcia-Bereguiain et al 2016, Wenner & Pekala 2022). Therefore, we are actually measuring the feature (synaptic strength) that we believe is homeostatically maintained, as opposed to spike rate, which we did not directly measure. Because we have not demonstrated a reliance of scaling on GABAR activation independent of spiking in the current study we did not mention it in the previous version of the manuscript. However, now we have added text on this in the discussion. See added text (lines 409-415).

2. (Related to 1): Do the authors have a sense of how the Clomeleon YFP/CFP ratios in this study (Fig. 2) translate to a specific measure of intracellular [Cl-]? The question is whether this change would be of a large enough magnitude to matter for excitability. In the Lindsly et al. 2014 paper (Fig. 3 of that study), there is a graph that estimates [Cl-] for a given ratio. If that graph were to apply to the ratios in this study, then the changes in chloride concentration would be in a narrow range (rising from ∼60 mM to ∼80 mM).

We are reticent to state an effect size of the change in Clomeleon ratios, as we do not know if the calibration studies would produce the exact same relationship to that of MNs (SPNs are significantly more superficial and this could influence ratios). Further, the changes in driving force and therefore current, would impact the excitability of the cell differently depending on the input resistance of the SPNs, which we do not know, but they are smaller and therefore likely have higher resistance. In our previous study the change in chloride was able to fully explain the change in mPSC amplitude. Regardless, we see the changes as an attempt to increase synaptic drive/strength, more so than excitability, and certainly this would occur. These changes would be particularly meaningful when considered across all of the GABAergic inputs to the cell. We have now added text briefly describing this in association with the response to point 1 (lines 283-286,409-415).

3. The idea of a stage-specific critical period is intriguing, but as written, it is a vague idea. It might be necessary to expound on this because - if this reviewer is interpreting correctly - the early timepoint observation is the main advance of this study beyond the similar observations in prior papers (e.g., Lidsly et al.).

Actually, the main advance of the study is that homeostatic plasticity, in this case GABAergic scaling, is expressed in the developing sympathetic nervous system, which has never been shown before. The stage at which GABAergic scaling is expressed is the same as in previous studies. We have tried to make this clearer (line 392-393).

So, beyond timing of when the authors looked, is there a reason to think that this form of plasticity contributes to an “early critical period” (Line 274)? This reviewer wonders why stage-specific homeostasis would be beneficial and how would a mode of synaptic scaling specifically contribute to an early critical period of ANS development - as opposed to being present throughout life to keep activity steady? The authors could expound on this in the Discussion section.

We have now included a more detailed description of how and why a critical period could influence the development of the sympathetic nervous system in the discussion. Generally, the point is that the sympathetic nervous system has been shown to be particularly vulnerable to early life perturbations (e.g. preterm birth) consistent with a critical period, which is typically associated with a plasticity that is expressed within the developing circuitry for a limited period. Indeed, we find that plasticity (in this case homeostatic) is expressed in the early developing circuitry of the sympathetic nervous system. We have added text better explaining these ideas (lines 409-424).

4. (Related to 3) Other studies examining homeostatic plasticity have shown that it can be subdivided into rapid or chronic forms in the nervous system. Do the authors have speculation on the timescale (and perdurance) of this form of homeostasis? This could also be highlighted in the Discussion.

We found evidence for chloride accumulation at 48 hours, so a slower form of homeostatic plasticity. Our results are very similar to our previous work in embryonic chick somatic motoneurons, which occur by 24 hours, but not by 12 hours of GABAergic blockade. Therefore, it is likely that a similar time course is expressed in SPNs and this is mentioned now in the discussion (line 393).

Minor points:

1. The author should clearly explain the conditions they refer to GABAergic as excitatory, simply because the idea of GABAergic signaling being excitatory will be a little counterintuitive to most readers.

We have added phrasing throughout which should help clarify the excitatory depolarizing nature of GABA at these early stages of development (lines 75, 219-221, 389-390).

2. This manuscript contains a lot of field-specific abbreviations that share letters or terminology with one another (SPN, ANS, SNS, etc.). A reader not in the immediate field might have difficulty with those abbreviations.

We agree, and now use autonomic nervous system in place of ANS, and sympathetic nervous system in place of SNS.

3. In the first section, the authors discuss chloride-mediated signaling in SPNs, where GABA is vital in mediating homeostasis. In the second part, there is a sudden shift to the cholinergic inputs from SPNs to PGNs. It is not clear to this reader exactly why one would necessarily predict (a priori) that the PGNs would execute a similar form of plasticity as the SPNs. Is it because they are both excitatory and in a linear circuit? What does it mean for the system that the PGNs seem not to show a homeostatic response?

We did not really have an a priori prediction about homeostatic plasticity in the PGNs, but because of the early vulnerability that we discuss above we wanted to test if we would see this form of plasticity in both the central and peripheral arm of the sympathetic nervous system. Further, it allows us to determine whether PGNs themselves might compensate for plasticity occurring in the SPNs or act as a simple relay. We tried to make that clearer in the manuscript (lines 294-296). In addition, we have attempted to clarify why PGNs may not express a compensatory plasticity at this stage (line 440).

4. Why would a shortened AP half-width predict activity in a compensatory direction? Quite the opposite, it might imply a briefer pulse for neurotransmission.

We agree with the reviewer and a similar comment of the first reviewer. Because of this we have removed any speculation of altered currents based on AP waveform.

Garcia-Bereguiain MA, Gonzalez-Islas C, Lindsly C, Wenner P. 2016. Spontaneous Release Regulates Synaptic Scaling in the Embryonic Spinal Network In Vivo. J Neurosci 36: 7268-82

Wenner PA, Pekala D. 2022. Homeostatic Regulation of Motoneuron Properties in Development In Vertebrate Motoneurons, ed. MJ O’Donovan, M Falgairolle, pp. 87-107. Cham: Springer International Publishing
