# Supplementary figures and images for: Plasticity in Preganglionic and Postganglionic Neurons of the Sympathetic Nervous System during Embryonic Development
Source: eNeuro. 2023 Nov 3;10(11):ENEURO.0297-23.2023. doi: 10.1523/ENEURO.0297-23.2023 (PMC10630925; doi:10.1523/ENEURO.0297-23.2023)

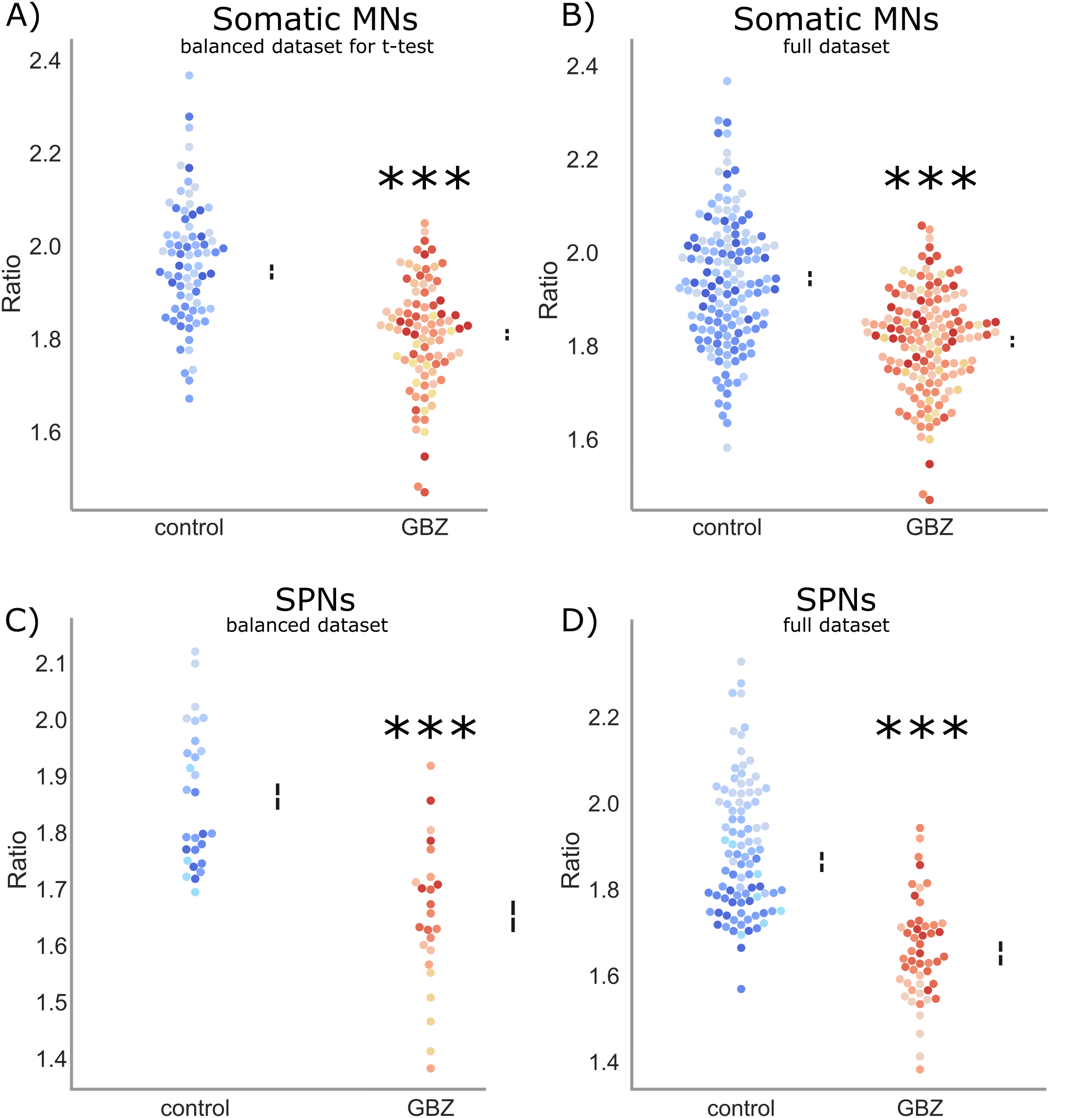

Supplement: Extended Data Figure 2-1 — Statistical confirmation of Clomeleon data. A, Motoneuron dataset showed significant difference between control and drug-treated groups. An independent, two sample t test was performed, restricting data to 10 cells per cord to handle uneven sampling between cords. This test revealed that ratio of YFP:CFP intensity was lower in cells from embryos treated with GBZ (1.81 ± 0.01) than from those treated with vehicle (H2O, 1.94 ± 0.02, t(175) = 6.7, p < 0.001). B, Full dataset is shown here, including every cell from each cord. This dataset is not suitable for a t test, however linear mixed effects modeling revealed a significant difference between groups was driven primarily by treatment group, not driven by cord umber or experiment date (p < 0.001). C, Analysis of SPNs also revealed a reduction in ratio in treated cells. An independent, two sample t test using four cells per cord revealed that FRET ratio of YFP:CFP were significantly lower in spinal cords from embryos treated with GBZ (1.65 ± 0.03) compared to those treated with H2O (1.86 ± 0.02, t(49) = 5.9, p < 0.001). D, Full dataset is shown here. As number of cells was imbalanced between cords, data was unsuitable for t test. Therefore, linear mixed effects test was performed on entire dataset. This test determined a significant difference between groups that was explained by treatment group alone, thus not driven the random factors of cord number or experiment date (p < 0.005). Bars represent standard error. Notches in bars represent mean. Download Figure 2-1, TIF file. [file enu-eN-NWR-0297-23-s01.tif]
